# Opioid switch from low dose of oral oxycodone to transdermal fentanyl matrix patch for patients with stable thoracic malignancy-related pain

**DOI:** 10.1186/1472-684X-13-46

**Published:** 2014-10-08

**Authors:** Seigo Minami, Takashi Kijima, Takeshi Nakatani, Suguru Yamamoto, Yoshitaka Ogata, Haruhiko Hirata, Takayuki Shiroyama, Taro Koba, Kiyoshi Komuta

**Affiliations:** 1 Department of Respiratory Medicine, Osaka Police Hospital, 10-31 Kitayama-cho, Tennoji-ku, Osaka 543-0035, Japan; 2 Department of Respiratory Medicine, Allergy and Rheumatic Diseases, Osaka University Graduate School of Medicine, 2-2 Yamada-oka, Suita, Osaka 565-0871, Japan

**Keywords:** Transdermal fentanyl matrix patch, Oxycodone, Opioid switch, Pain, Thoracic malignancy

## Abstract

**Background:**

The effectiveness and safety of switch from oral oxycodone to fentanyl patch is little known. Here, we investigated if early phase opioid switch from low dose of oral oxycodone to transdermal fentanyl matrix patch provided any benefits for patients with thoracic malignancy and stable cancer-related pain.

**Methods:**

This open-label two-centered prospective study enrolled patients with thoracic malignancy suffering persistent malignancy-related pain with numeric rating scale of pain intensity ≤ 3 which had been controlled by oral oxycodone ≤ 20 mg/day. Eligible patients switched from oral oxycodone to 12.5 μg/h of transdermal fentanyl matrix patch. The dose was allowed to be titrated upwards every 3 day by 25-50%, except for the first increase from 12.5 μg/hr to 25 μg/hr,until achieving adequate pain control. The data on patients’ global assessment scores measured on a five-step scale, an 11-point numeric rating scale of pain intensity, the severity of adverse effects using a four-point categorical rating scale, and the Epworth sleepiness scale questionnaire were collected for 15 days.

**Results:**

Forty-nine eligible patients were analyzed. Overall patients’ satisfaction score significantly improved from day 1 (2.7 ± 0.9) to day 15 (2.3 ± 0.9) (*p* < 0.05), and 90% and 78% of patients remained to receive the minimum dose of fentanyl patch on day 8 and 15 from the opioid switch. There was a significant difference in sleepiness throughout the study period, though no difference was detected in pain intensity and other adverse effects.

**Conclusion:**

Transdermal fentanyl matrix patch is an alternative analgesic option for a stable cancer pain in patients with thoracic malignancies.

## Background

Cancer-related pain is the most important concern in patients suffering cancer. More than 80% of patients with advanced disease experience cancer-related pain caused largely by direct tumor invasion [1]. Cancer-related pain often disturbs activities of daily living and deteriorates quality-of-life [2].

Opioids are the mainstay of analgesic therapy for moderate to severe cancer-related pain, according to the current guidelines [1,3]. Oxycodone, a semi-synthetic opioid analgesic, has become a cornerstone in symptom management of cancer-related pain [4-6]. Controlled-release of oxycodone is often administered as the first-line strong opioid, typically at an initial dose of 5-10 mg every 12 hours in opioid-naïve patients, when non-steroidal anti-inflammatory drugs (NSAIDs) and weak opioids are ineffective.

On the other hand, the high lipid solubility and low molecular weight of fentanyl is suitable for transdermal administration. In 2002, a transdermal therapeutic system (TTS) for fentanyl incorporating a gel reservoir technology was first marketed as the Durotep^®^Patch (Janssen-Pharma, Japan). In 2008, a transdermal fentanyl matrix patch (Durotep^®^MT patch, Janssen-Pharma, Japan), a new TTS that contains fentanyl dissolved in the adhesion layer, has become available in Japan. The smallest size of the matrix patch, with fentanyl release rates of 12.5 μg/h, makes it possible to switch from lower dose of other opioid and then gradually titrate to the effectual dose [7]. Fentanyl may has not only a powerful analgesic effect, but advantage of less frequent and milder adverse effects such as nausea, vomiting, constipation and sleepiness [8-12]. Compared with oral morphine, fentanyl may have advantage especially in constipation, but there are concerns over methodological problems of constipation assessment. In a crossover trial, constipation was less frequent under transdermal fentanyl than under oral morphine, though the assessment methods of constipation remained unclear [9]. In another comparison study, the use of laxatives given by nurses was investigated instead of assessment of constipation and shown to be less frequent in fentanyl [12]. Two meta-analyses [13,14] and a systematic review [15] also indicated a significant reduction in constipation for fentanyl patch compared with oral morphine. In contrast, another prospective comparison study showed that transdermal opioids of fentanyl and buprenorphine had no benefit over controlled release oral hydromorphone in gastrointestinal symptoms including constipation [16]. However, the evidence on an advantage of fentanyl patch over oral opioids is low level, because the trials regarding fentanyl patch were limited, not blinded and of low methodological quality [3].

Opioid switch is a therapeutic maneuver aiming at improvement of analgesic response and reduction of adverse effects [17]. The maneuver includes change to different medication using the same administration route, alteration of administration route maintaining the current medication, or both. Fentanyl patch is the most favorable candidate of opioid switch from oral opioids because of less adverse effects and easier administration route [9,10,18,19]. Switch from oral morphine to transdermal fentanyl patch was shown as an effective and safe method [9,10], though the drop-out rate with fentanyl was higher than that with morphine in a crossover trial [9]. In contrast, little is known about the benefits of switch from oral oxycodone to fentanyl patch.

In this study, we investigated if early phase opioid switch from low dose of oral oxycodone to transdermal fentanyl matrix patch provided any benefits for patients with stable cancer-related pain.

## Methods

### Patient eligibility

Patients with thoracic malignancy-related pain were recruited in Osaka Police Hospital and Osaka University Hospital. The patients who met all the following criteria were eligible; 1) confirmed diagnosis of thoracic malignancy, 2) age ≥ 20 years, 3) persistent malignancy-related pain refractory to non-steroidal anti-inflammatory drugs (NSAIDs) or acetaminophen, 4) numeric rating scale (NRS) for pain ≤ 3 for at least two consecutive days by low dose (20 mg/day or less) of oral oxycodone (Oxycontine^®^, Shionogi-Pharma, Japan). Exclusion criteria were as follows; 1) prior history of allergy or hypersensitivity to opioids, 2) prior history of opioid abuse, 3) clinically significant cardiac, renal or hepatic insufficiency, 4) increased intracranial pressure or impaired cognitive function, 5) active and extensive skin disease precluding application of the transdermal delivery system, 6) persistent fever of 40 degrees Celsius or over, 7) pregnancy, lactation and suspicion of being pregnant, 8) prior use of any opioid-antagonists within 2 days before switching to fentanyl patch. Concomitant treatments such as chemotherapy and radiotherapy at the enrollment were accruable.

### Study design and treatment

All patients recorded the following data daily for 15 days of fentanyl patch treatment; 1) pain intensity using an 11-point NRS (scales 0–10), 2) the severity of adverse effects (e.g., nausea, vomiting, constipation and sleepiness) using a four-point categorical rating scale (none = 0, not hard = 1, hard but endurable = 2 and unendurably hard = 3), 3) the number of defecation per day, 4) the Epworth sleepiness scale (ESS) questionnaire except for the question on sleepiness during driving a car because strong opioids are not permitted for drivers in Japan, 5) the frequencies of immediate-release oxycodone (Oxinorm^®^, Shionogi-Pharma, Japan) medications per day. Time of recording diary was arbitrary. The diaries were collected after 15 days of fentanyl patch treatment. The patients' global assessment scores were also rated using a five-step scale (very satisfied = category 1, satisfied = 2, neither satisfied nor dissatisfied = 3, dissatisfied = 4 and very dissatisfied = 5) [20] on days 1, 8 and 15. Oral oxycodone was switched to 12.5 μg/h of transdermal fentanyl matrix patch. In the morning on the switch day (day 1), a fentanyl patch (2.1 mg/3 days) was applied at the same time of the oral intake of the last controlled-release oxycodone dose. Rescue immediate-release oxycodone dose to relieve the breakthrough pain was adjusted according to the dose of transdermal fentanyl patch. The dose of transdermal fentanyl patch was allowed to be titrated upwards every 3 days by 25-50%, except for the first increase from 2.1 mg/patch (12.5 μg/hr) to 4.2 mg/patch (25 μg/hr), until patients could achieve adequate pain control. Transdermal fentanyl patch treatment was continued for 15 days (5 replacements). Neither addition nor dose change of other supplementary analgesics was permitted during the study.

The primary endpoint in this study was patients' global assessment scores. Secondary endpoints were pain intensity, adverse effects and use of rescue oxycodone.

The study protocol was approved by the Institutional Review Board of Osaka University Hospital and the Osaka Police Hospital ethics committee, adhered to the principles outlined in the Guideline for Good Clinical Practice (January 1997) and Declaration of Helsinki (1996), and registered as UMIN000011067. Written informed consent was obtained from all patients before commencement of the study.

### Statistical analysis

We expected that 85% of patients would give the global assessment scores 1 to 3, on the basis of the data from a previous Japanese clinical trial of Durotep^®^MT patch reporting that the patients’ satisfaction was 89.4% [20]. The estimated number of patients for analysis was 49 with a confidence interval of 95% and β-error of 10%. Given the possibility of deviation from assessment, 60 patients were necessary.

Listwise deletion was adopted in cases in which we missed data at opioid switch (day 1). Patients who dropped out without completion of 15-day patch treatment were also deleted from analysis. Missing data at days 8 and 15 from opioid switch were handled using last observation carried forward (LOCF) imputation technique.

The data for normally distributed continuous variables, discrete variables, and categorical variables are expressed as the mean ± standard deviation (SD), median with range, and frequency. Friedman test, with a post-hoc Steel test, was used to compare longitudinal changes in numeric and categorical rating scales and ESS questionnaire scores. Significance was set at *p* < 0.05. All statistical analyses were performed using Statcel statistical package (Statcel3; OMS Inc., Tokorozawa, Japan).

## Results

Although enrollment to this study was planned to complete within 2 years, only 50 patients were accrued for 4 years, despite of 2-year extension of the study period. Therefore, this study was terminated in December 2012 because of delayed accrual. The final accrual did not reach 60 patients, the preplanned sample size.

From February 2009 to December 2012, a total of 50 eligible patients switched opioids. Almost all patients completed 15 days of fentanyl matrix patch treatment except one patient who suddenly died of cancer progression on the 13rd day from opioid switch. During the study period, 51% and 24% of patients concomitantly received chemotherapy and radiotherapy, respectively. The baseline characteristics are shown in Table 1.

**Table 1:** Patients’ characteristics (N = 49)

Sex	
Male/Female	43/6
Age (years-old)	69.0 ± 6.9
Body mass index (kg/m^2^)	20.4 ± 4.9
Cancer (Histology)	
Lung Cancer (Ad/SQ/SCLC/Others)	48 (26/14/4/4)
Mesothelioma	1
Stage	
IIB/IIIA/IIIB/IV^a^	1/4/7/37^a^
ECOG PS	
0-1/2/3/4	28/13/5/3
Pain location^b^	
Neck-shoulder/Upper limb/Chest/Hypochondrium/Lower back/Glutaeus to thigh	
	4/2/32/1/7/5
Pain causes^b^	
Distant metastasis (bone)/Invasion/Pleural dissemination	
	22 (17)/18/11
Duration of cancer pain (months)	
mean ± SD	4.3 ± 4.4
median (range)	2 (0.27–18)
Duration of controlled-release oxycodone administration (months)	
mean ± SD	2.0 ± 4.3
median (range)	0.6 (0.1 - 19.7)
Fentanyl patch treatment site	
day 1	
Hospitalized/Outpatient	40/9
day 8	
Hospitalized/Outpatient	32/17
day 15	
Hospitalized/Outpatient	22/27
Concomitant systemic chemotherapy	
Yes/No	25/24
Concomitant radiotherapy	
Yes/No	12/37

Ad, Adenocarcinoma; SQ, Squamous cell carcinoma; SCLC, Small cell lung carcinoma; ECOG PS, European Clinical Oncology Group Performance status; SD, standard deviation.

^a^including a case with malignant mesothelioma in c-stage IV.

^b^There were two overlapping.

Overall patients’ satisfaction was significantly different throughout the study period (Friedman test; *p* < 0.01). The proportion of patients in category 1 or 2 (‘very satisfied’ or ‘satisfied’) for the patient's global assessment score increased from 43% on day 1 to 63% on day 8 and 61% on day 15, while the proportion of patients in category 1–3 (‘very satisfied’, ‘satisfied’ or ‘neither satisfied nor dissatisfied’) hardly changed from 87% on day 1 to 91% on day 15 (Table 2).

**Table 2:** **Change of patients’ global assessment scores (N = 46 **^**a**^**)**

	**Day 1**	**Day 8**	**Day 15**
mean ± SD	2.7 ± 0.9	2.4 ± 0.9	2.3 ± 0.9
median (range)	3 (1 - 5)	2 (1 - 5)	2 (1 - 5)
*vs.* day 1^b^		n.s.	n.s.
Patients’ distribution (N)			
1. Very satisfied	2	5	9
2. Satisfied	18	24	19
3. Neither	20	12	14
4. Dissatisfied	4	4	3
5. Very dissatisfied	2	1	1

^a^Three patients were excluded from analysis because of missing data throughout all the three observation points.

^b^Post-hoc nonparametric multiple comparison analysis was performed using Steel method after significant difference in assessment scores across multiple points was detected by Freidman test (*p* < 0.01).

n.s.; not significant (*p* > 0.05).

SD; standard deviation.

Oxycodone doses on day 1 differed from 10 to 20 mg/day. Fentanyl matrix patch dose remained 12.5 μg/h in 44 patients (90%) on day 8 and 38 patients (78%) on day 15, and increased up to more than 25 μg/h in 10 patients (20%) during the study period (Table 3). Six, eight and six patients felt pain with NRS ≥ 4 at day 1, 8 and 15, respectively. Among them, one, two and two patients were under outpatient care at day 1, 8 and 15, respectively. There was no significant difference between 3 measurement days in pain intensity, rescue dose of oxycodone and adverse effects except sleepiness (Friedman test, *p* = 0.01) (Tables 4 and 5 and Additional file 1: Table S1).

**Table 3:** Dose of oral oxycodone and transdermal fentanyl matrix patch at day 0, 8 and 15 (N = 49)

	**Day 1**		**Day 8**		**Day 15**	
**Opioids**	**Oral oxycodone**		**Fentanyl patch**		**Fentanyl patch**	
Dose						
Mean ± SD	14.7 ± 4.1 mg/day		13.7 ± 4.8 μg/h		15.5 ± 6.0 μg/h	
Patients’ distribution (N)	10 mg/day	18	12.5 μg/h	44	12.5 μg/h	38
	15 mg/day	16	18.8 μg/h*	1	18.8 μg/h*	1
	20 mg/day	15	25.0 μg/h	3	25.0 μg/h	9
			37.5 μg/h	1	37.5 μg/h	1

All patients were switched from oral oxycodone to 12.5 μg/h of fentanyl matrix patch at day 1.

*Half-side application procedure of fentanyl matrix patch was not defined in our protocol.

SD; standard deviation.

**Table 4:** Change of numeric rating scale (NRS) of pain intensity and use of rescue immediate-release oxycodone

	**Day 1**	**Day 8**	**Day 15**	***p*-value**^**a**^
NRS pain intensity (N = 49)				0.15
Mean ± SD	2.2 ± 1.4	2.1 ± 1.5	1.9 ± 1.4	
Median (range)	2 (0 - 6)	2 (0 - 7)	2 (0 - 6)	
NRS 0 – 3 / ≥ 4	43/6	41/8	43/6	
Immediate-release oxycodone (mg / day) (N = 48)^b^				0.36
Mean ± SD	1.9 ± 2.2	2.7 ± 3.5	2.2 ± 2.7	
Median (range)	2.5 (0 - 10)	2.5 (0 - 15)	1.75 (0 - 10)	

SD; standard deviation.

^a^Friedman test.

^b^One patient was excluded from analysis of rescue use because of missing data throughout all the three observation points. The remaining 48 patients completed data collection.

**Table 5:** Adverse events

	**N**	**Day 1**	**Day 8**	**Day 15**	***p*-value**^**a**^
Sleepiness	45				0.004
Mean ± SD		1.2 ± 0.7	0.9 ± 0.6	0.73 ± 0.8	
Median (range)		1 (0 - 3)	1 (0 - 2)	1 (0 - 3)	
*vs.* day 1^b^			n.s.	<0.05	
Nausea	49				0.87
Mean ± SD		0.4 ± 0.8	0.4 ± 0.7	0.3 ± 0.6	
Median (range)		0 (0 - 3)	0 (0 - 3)	0 (0 - 2)	
Vomit	49				0.66
Mean ± SD		0.1 ± 0.6	0.2 ± 0.6	0.1 ± 0.5	
Median (range)		0 (0 - 3)	0 (0 - 3)	0 (0 - 2)	
Constipation	48				0.08
Mean ± SD		0.5 ± 0.7	0.3 ± 0.5	0.3 ± 0.5	
median (range)		0 (0 - 3)	0 (0 - 2)	0 (0 - 2)	
Defecation number (/ day)	49				0.85
Mean ± SD		1.1 ± 1.0	1.0 ± 0.8	1.0 ± 0.7	
Median (range)		1 (0 - 4)	1 (0 - 4)	1 (0 - 4)	
Epworth Sleep Scale	49				0.96
Mean ± SD		6.3 ± 4.0	6.1 ± 4.2	6.0 ± 4.2	
Median (range)		6 (0 - 16)	5 (0 - 18)	5 (0 - 19)	

SD; standard deviation, n.s.; not significant (*p* > 0.05).

^a^Friedman test.

^b^Post-hoc nonparametric multiple comparison analysis was performed using Steel method after significant difference in assessment scores across multiple points was detected by Freidman test (*p* = 0.01).

## Discussion

This is the first study to evaluate opioid switch directly from low dose of oral oxycodone to fentanyl matrix patch in patients with malignancy-related pain.

Another Japanese study also investigated opioid switch to 12.5 μg/h of fentanyl matrix patch from various opioids. This study was different from ours in the following three points; 1) including various kinds of prior opioid; oral oxycodone < 30 mg/day (69.4%), intravenous fentanyl injection < 0.3 mg/day (1.2%) and oral, transanal or intravenous morphine (29.4%), 2) recruiting patients with various types of primary cancers, including 34% of respiratory cancer, 3) assessing patients’ global pain assessment only at day 10, during third patch application, or the day of protocol withdrawal [20]. We focused on switch only from oral oxycodone ≤ 20 mg/day, recruited only patients with thoracic malignancy, and compared patients’ global assessments among 3 measurement days.

The most important finding of our study was that patients’ satisfaction was improved by opioid switch from oral oxycodone to fentanyl patch. More than 80% of patients did not feel dissatisfied in the patients' global assessment scores, which met the primary endpoint. Contrast to no difference in ESS score, sleepiness was significantly improved by opioid switch. Moreover, favorable changes were conceivably noted in constipation, though the improvement in constipation did not reach statistical significance. In the four-point categorical rating scale of sleepiness (scales 0 - 3), the number of patients with scale 0 increased from 6 on day 1 up to 19 on day 15, while that with scale 1 decreased from 29 down to 20. In the assessment of constipation, all 5 patients with scale 2 or 3 on day 1 improved to scale 0 or 1 on day 15, while one patient with scale 0 on day 1 deteriorated into scale 2 on day 15 (Additional file 1: Table S1). These changes were similar to those in the study by Miyazaki *et al*., in which the rate of ‘very satisfied’ and ‘satisfied’ increased over time after opioid switch to fentanyl patch [20].

The second important finding was that conversion rate from oral oxycodone to fentanyl patch was not uniform. In our study, various doses of oral oxycodone from 10 to 20 mg/day were switched uniformly to 12.5 μg/h of fentanyl patch. Not a few patients felt pain of NRS ≥ 4 and needed increase of fentanyl patch thereafter. The equivalent dose of fentanyl patch to oral oxycodone was not definite in our study, though an initial conversion from 10-20 mg/day of oral oxycodone to 12.5 μg/h of fentanyl patch seems safe and reasonable. Thus, we have to pay careful attention to switch from oral oxycodone to fentanyl patch for fear of insufficient pain control or overdose.

Our study included some limitations. First, our study was not blinded. A bias derived from different formulations was possible. Second, we used an unvalidated tool to assess constipation, thereby this adverse event might be either underestimated or overestimated. Third, more than half patients concomitantly received other cancer treatment such as chemotherapy or radiotherapy. Because these concomitant treatments possibly influenced cancer pain, the true analgesic power of fentanyl patch was uncertain in those patients. Considering the poor prognosis of advanced lung cancer, we could not forbid any other cancer treatments during the study period. Fourth, the day 1 at opioid switch might not be appropriate as a baseline assessment for comparison with day 8 and 15. In addition, we should have standardized the timing of diary record. Although all patients had reported pain of NRS ≤ 3 until the previous day of opioid switch, six patients suffered from pain of NRS level ≥ 4 at the day of opioid switch (on day1). Five of these 6 patients converted opioids on day1 in our hospitals, and recorded their diaries on day 1 before or at the same time as the first application of fentanyl patch. Because we did not know when the remaining one outpatient had written diaries on day 1 at home, this patient might have severer pain than usual by the confusion from oral oxycodone to fentanyl patch. Fifth, we did not grasp the precise number of ineligible patients who dropped out during oxycodone treatment due to uncontrollable pain by 20 mg/day of oral oxycodone. Thus, we failed to clarify the proportion of eligible patients for this opioid switch maneuver in all patients with thoracic malignancy-related pain requiring strong opioid. Based on the study limitations described above, we are considering a cross-over trial comparing in pain control and adverse severity between fentanyl patch and oral opioid for cancer-related pain, instead of a randomized blinded controlled trial. Our study group is still too small to conduct such a high level of trial.

## Conclusion

Transdermal fentanyl matrix patch is an alternative analgesic option for a stable cancer pain in patients with thoracic malignancies.

## Competing interest

The authors declare that they have no competing interests.

## Authors’ contribution

TK designed the study. SM and TK analyzed the data and wrote the manuscript. All authors accrued and managed patients, collected data, read and approve this manuscript, and agree to its submission.

## Pre-publication history

The pre-publication history for this paper can be accessed here:

http://www.biomedcentral.com/1472-684X/13/46/prepub

## Supplementary Material

Additional file 1: Table S1 The patients’ distribution of adverse events. Click here for file
