# Supplementary material for: Opioid switch from low dose of oral oxycodone to transdermal fentanyl matrix patch for patients with stable thoracic malignancy-related pain
Source: BMC Palliat Care. 2014 Oct 8;13:46. doi: 10.1186/1472-684X-13-46 (PMC4195703; doi:10.1186/1472-684X-13-46)
Supplement: Additional file 1: Table S1 — The patients’ distribution of adverse events. [file 1472-684X-13-46-S1.docx]

Supplemental Table1. The patients’ distribution of adverse events

|  | N | Score | Day 1 | Day 8 | Day 15 |
| --- | --- | --- | --- | --- | --- |
| Sleepiness | 45 | 0  1  2  3 | 6  29  7  3 | 11  29  5  0 | 19  20  5  1 |
| Nausea | 49 | 0  1  2  3 | 36  8  3  2 | 35  11  2  1 | 38  7  4  0 |
| Vomit | 49 | 0  1  2  3 | 46  1  0  2 | 44  3  1  1 | 44  3  2  0 |
| Constipation | 48 | 0  1  2  3 | 31  12  4  1 | 35  12  1  0 | 37  10  1  0 |
| Defecation rate (times/ day) | 49 | 0  1  2  3  4 | 13  23  9  1  3 | 13  25  10  0  1 | 9  33  5  1  1 |
| Epworth Sleep Scale | 50 | 0-4  5-8  9-12  12-16  ≥ 17 | 20  16  8  5  0 | 21  16  6  5  1 | 21  18  4  5  1 |
